# Downregulation of circulating exosomal miR-638 predicts poor prognosis in colon cancer patients

**DOI:** 10.18632/oncotarget.19689

**Published:** 2017-07-29

**Authors:** Shushan Yan, Guangwang Dang, Xiaoyu Zhang, Chengwen Jin, Lang Qin, Yugang Wang, Min Shi, Haijin Huang, Quanhong Duan

**Affiliations:** ^1^ Department of Gastrointestinal and Anal Diseases Surgery, The Affiliated Hospital of Weifang Medical University, Weifang, China; ^2^ Department of Outpatient, People's Hospital of Zoucheng, Zoucheng, China; ^3^ Division of Gastrointestinal Surgery, Department of General Surgery, The Affiliated Huai'an Hospital of Xuzhou Medical University and The Second People's Hospital of Huai'an, Huai'an, China; ^4^ Functional Laboratory, Clinical Medicine College of Weifang Medical University, Weifang, China; ^5^ Department of Image, The First Affiliated Hospital of Nanjing Medical University, Nanjing, China; ^6^ Department of Gastroenterology, Shanghai Tongren Hospital, Shanghai Jiao Tong University School of Medicine, Shanghai, China; ^7^ Department of General Surgery, Hongze District People's Hospital, Hongze, China

**Keywords:** colon cancer, exosomes, microRNA-638, prognosis

## Abstract

Exosome-encapsulated microRNAs have been recognized as novel and stable biomarkers for cancer. However, little is known about the role of exosomal microRNAs in colon cancer. In the present study, we investigated the expression of serous exosomal microRNA-638 (miR-638) and its prognostic effect in patients with colon cancer. Serous exosomal samples were assayed by quantitative real-time PCR. Kaplan-Meier analysis was adopted to determine the overall survival (OS) and disease-free survival (DFS) of colon cancer patients. Cox regression analysis was applied to assess the potential association between serous exosomal miR-638 and clinicopathological factors of colon cancer patients. MiR-638 was significantly reduced in serum exosomes of colon cancer patients compared with healthy controls. Decreased level of serous exosomal miR-638 was more significant in colon cancer patients at later TNM stage or with liver metastasis. Kaplan-Meier analysis showed that colon cancer patients with reduced level of serous exosomal miR-638 had poor OS and DFS. In addition, the Cox regression analysis suggested serous exosomal miR-638 was a prognostic factor for colon cancer independent of TNM stage and liver metastasis. In conclusion, serous exosomal miR-638 is a useful biomarker for the prediction of colon cancer prognosis.

## INTRODUCTION

Colon cancer is one of the leading causes of cancer-associated death in the world [1]. To the best of our knowledge, genetics, way of life, and colon adenoma are closely related to the pathogenesis of this disease. Accumulating epigenetic data have implicated that colon cancer is a deadly disease attributed to interactions between multiple genes, such as cumulative mutations and aberrant methylation of certain genes, such as M2 isoform of pyruvate kinase (PKM2) and angiotensin II receptor, type 1 (AGTR1) [2, 3]. Colon cancer cells grow uncontrollably due to the activation and overexpression of oncogenes and the mutation and deletion of tumor suppressor genes [2]. In spite of progress in the prevention and early detection of colon cancer, the 5-year survival rate of colon cancer remains lower, particularly for those patients at advanced stages [4, 5]. As a result, identification of prognostic factors can improve the efficacy of current treatments and the life quality of colon cancer patients.

In recent years, circulating exosomal microRNAs have been found to play critical roles in regulating the development of colon cancer [6, 7]. As non-coding RNA molecules, microRNAs are small 19 to 25 nucleotides of RNA, which engage in the cell differentiation, apoptosis, and cell cycle progression [8]. MicroRNAs can regulate the expression of targeted genes at the post transcriptional level. The expression of microRNAs is highly conserved with spatial and temporal expression specificity [9]. As a result, different diseases may have diverse specific expression profiles of microRNAs. In addition, the same disease may also have different organ or tissue specific expression profiles of microRNAs. Therefore, specific microRNA expression profiles may be used as biomarkers in patients with colon cancer. In the past few years, tumor derived exosomal microRNAs have been identified as a new group of circulating tumor markers and potential therapeutic targets by regulating tumor angiogenesis, tumor cell growth, and invasion [10, 11]. In our previous study, we have identified a specific circulating exosomal microRNAs expression profile in serum from colon cancer patients. MicroRNA-638 (miR-638) is one of the most significantly decreased microRNAs in serum exosomes of patients with colon cancer. However, little is known about the prognostic value of circulating exosomal miR-638 in colon cancer. The aim of this study is to investigate the association between circulating exosomal miR-638 and the prognosis of colon cancer patients.

## RESULTS

### Characteristics of the study population

Characteristics of all participants were presented in Table 1. Overall, 108 male and 84 female patients with colon cancer were recruited in our study. The mean age at diagnosis of all colon cancer patients was 57.8 ± 11.2 years, and no statistically significant difference for the mean age at diagnosis was observed between colon cancer patients with high or low levels of serum exosomal miR-638 (the miR-638^high^ group: 57.5 ± 11.0 years; the miR-638^low^ group: 58.0 ± 10.8 years) (Table 1). There was no statistical significance between the two groups with regard to age, gender, tumor stage, tumor differentiation status, and vascular filtration.

**Table 1:** Characteristics of all patients with colon cancer

Factors	miR-638 ^high^ (n=64)	miR-638 ^low^ (n=128)	*P*-value
Age, n (%)			1.00
<58 years	38 (59.4)	76 (59.4)	
≥58 years	26 (40.6)	52 (40.6)	
Gender, n (%)			0.53
Female	26 (40.6)	58 (45.3)	
Male	38 (59.4)	70 (54.7)	
TNM stage, n (%)			0.45
I	12 (18.7)	25 (19.5)	
II	9 (14.1)	24 (18.8)	
III	33 (51.6)	65 (50.8)	
IV	10 (15.6)	14 (10.9)	
Tumor differentiation status, n (%)			0.91
High	46 (71.9)	93 (72.7)	
Low	18 (28.1)	35 (27.3)	
Vascular infiltration, n (%)			0.76
Yes	9 (14.1)	16 (12.5)	
No	55 (85.9)	112 (87.5)	

### Expression and its clinicopathological significance of serous exosomal miR-638 in colon cancer patients

**Figure 1:**
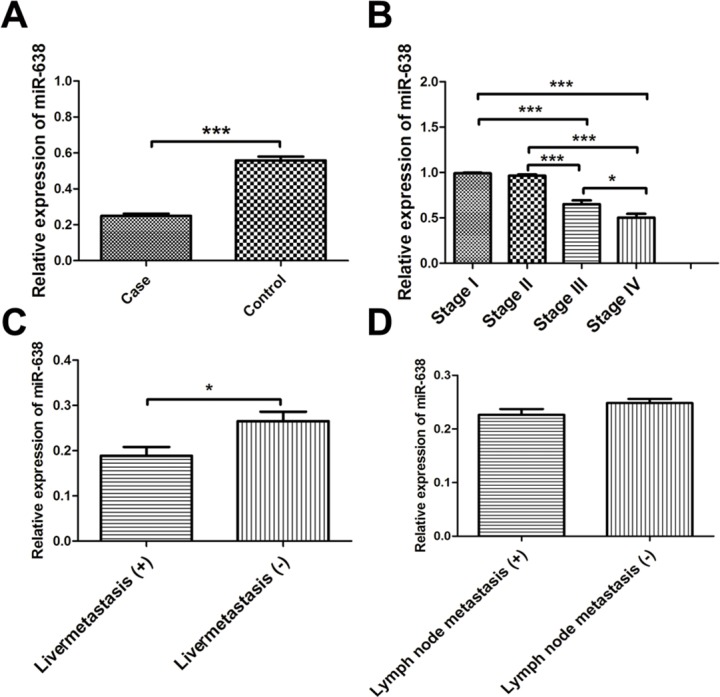
**(A)** Deceased levels of serous exosomal miR-638 in patients with colon cancer. **(B)** Expression of serous exosomal miR-638 in colon cancer patients at different TNM stage. **(C)** Expression of serous exosomal miR-638 in colon cancer patients with or without liver metastasis. **(D)** Expression of serous exosomal miR-638 in colon cancer patients with or without lymph node metastasis. *, *P* < 0.05; ***, *P* < 0.001.

We found miR-638 was significantly deceased in serum exosomes of patients with colon cancer compared with healthy controls (Figure 1A). Besides, reduced expression of serous exosomal miR-638 was more significant in colon cancer patients at later TNM stage (Figure 1B). Moreover, levels of serous exosomal miR-638 were much lower in patients with liver metastasis but not lymph node metastasis (Figure 1C and 1D).

### Association between serous exosomal miR-638 and the OS and DFS of colon cancer patients

**Figure 2:**
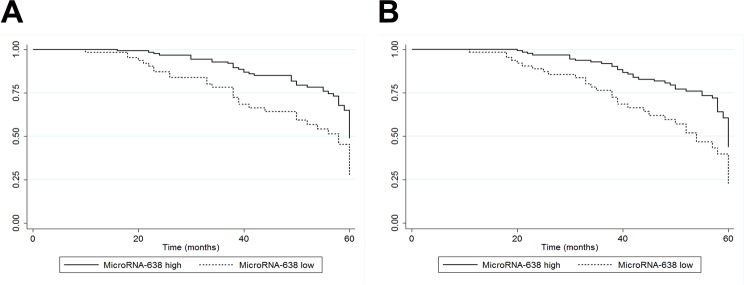
**(A)** Kaplan-Meier survival analysis of OS (*P* < 0.01). **(B)** Kaplan-Meier survival analysis of DFS (*P* < 0.01).

Kaplan-Meier analysis showed that colon cancer patients with low levels of serous exosomal miR-638 had significantly shorter OS and DFS than those with high levels of serous exosomal miR-638 (*P_OS_* < 0.01, *P_DFS_* < 0.01; Figure 2A and 2B). The Cox regression analysis suggested that low levels of serous exosomal miR-638 was associated with poor prognosis in colon cancer patients (Table 2). Colon cancer patients at later TNM stage or with liver metastasis or low levels of exosomal miR-638 in serum had poor OS (Table 2). Additionally, low level of serous exosomal miR-638 was also related to poor prognosis in colon cancer patients independent of other significant factors, including TNM stage and liver metastasis (Table 2).

**Table 2:** Cox regression analysis for the association between serum exosomal miR-638 and OS of colon cancer patients

Factors	Univariate analysis		Multivariate analysis	
	HR(95%CI)	*P*-value	HR(95%CI)	*P*-value
Age (≥58 years)	1.23(0.78-1.93)	0.367	1.15(0.73-1.82)	0.542
Gender (Male)	1.04(0.66-1.63)	0.853	1.00(0.64-1.57)	0.999
TNM stage (III/IV)	2.25(1.30-3.90)	0.004	3.13(1.70-5.75)	<0.001
Tumor differentiation grade (Low)	1.47(0.92-2.34)	0.105	2.28(1.37-3.78)	0.001
Vascular infiltration	0.76(0.33-1.76)	0.528	0.99(0.41-2.41)	0.987
Lymph node metastasis	1.14(0.56-2.43)	0.278	1.87(1.04-3.49)	0.345
Liver metastasis	2.76(1.29-3.98)	0.007	3.13(1.70-5.75)	0.009
Low levels of serum exosomal miR-638	2.01(1.28-3.17)	0.003	1.90(1.20-3.91)	0.006

## DISCUSSION

Exosomes are nano-sized vesicles with a diameter of 40-100nm in the biofilm structure. They widely distribute in saliva, blood, and milk. Exosomes consist of proteins, lipids, mRNAs, long non-coding RNAs, and microRNAs that can be delivered to different cells, and thus mediate intercellular communication. Increasing evidence has suggested exosomes are involved in regulating the immune response, antigen presentation, cell differentiation, cell metabolism, tumor invasion and other biological processes [12, 13]. Recent evidence has indicated that exosomes mediate interactions between cancer cells and the microenvironment [14, 15]. Exosomes are the natural carriers. Exosomes-encapsulated microRNAs can function as intracellular and extracellular microRNAs by cell to cell communications. However, the extracellular microRNAs can be used for diagnostic and therapeutic targets, in that they can be secreted as signaling molecules by certain cells. Specific microRNAs transferred by serum exosomes may play a vital role in the pathogenesis of malignant tumors. Therefore, exosomal microRNAs merit consideration as prognostic markers, diagnostic markers and potential therapeutic targets. Although microRNAs have been widely investigated in cancer development and progression, the role of circulating exosomal microRNAs and their underlying regulatory mechanisms in colon cancer are largely unknown. Previously, we have screened the specific exosomal microRNAs profile in serum from patients with colon cancer, and found that miR-638 was one of the most significantly reduced exosomal microRNAs in serum of patients. Besides, miR-638 was significantly decreased in serum exosomes of colon cancer patients at later TNM stage (II/III/IV). Moreover, low levels of serous exosomal miR-638 were associated with increased risk of liver metastasis in patients with colon cancer. Based on these findings, we hypothesized that the expression of miR-638 in serum exosomes might influence the prognosis of colon cancer patients. Therefore, we performed a postoperative follow-up in 192 histologically diagnosed patients with colon cancer, and found that decreased levels of serous exosomal miR-638 in colon cancer patients were indeed related to poor prognosis. Patients with low levels of miR-638 in serum exosomes had much shorter OS and DFS than those with high levels of serous exosomal miR-638. In addition, serous exosomal miR-638 was a prognostic factor for colon cancer independent of TNM stage and liver metastasis. Taken together, low levels of serous exosomal miR-638 predicted poor prognosis in patients with colon cancer.

Dysregulation of miR-638 has been described in a number of cancers, such as hepatocellular carcinoma, gastric carcinoma, and colorectal carcinoma [16–18]. Nonetheless, the expression levels of miR-638 vary with diverse locations and cancers. Significantly increased levels of miR-638 were observed in hepatocellular carcinoma tissues compared with normal liver tissues [19], while decreased levels of miR-638 were found in tumor tissues of hepatocellular carcinoma patients in contrast to their paired non-cancerous tissues [16]. The inconsistent findings may be attributed to different sample size, source of controls and individual variations. Although low level of miR-638 in certain tumor tissues can predict poor prognosis, including hepatocellular carcinoma and colorectal cancer [3, 19], little is known about its prognostic value of circulating exosomal miR-638 in cancer. In the current study, we firstly found that serous exosomal miR-638 is downregulated in colon cancer patients. Besides, low levels of serous exosomal miR-638 were associated with TNM stage and live metastasis, predicting potential role of exosomal miR-638 in the progression of colon cancer. Therefore, serous exosomal miR-638 may serve as a novel and noninvasive biomarker for the prognosis of colon cancer. In addition, serous exosomal miR-638 was a new prognostic factor for colon cancer independent of age, gender, TNM stage, tumor differentiation status, vascular infiltration, lymph node metastasis, and liver metastasis.

The underlying molecular mechanisms by which circulating exosomal miR-638 affects colon cancer are important. Nevertheless, the exact role of circulating exosomes in cancer remains largely unknown, except for their effect in mediating intercellular communication via the release of vesicle contents, such as circulating non-coding RNAs, proteins and lipids. Ren Y and the colleagues have recently reported that miR-638 was aberrantly expressed in cancer tissues and could promote autophagy and malignant phenotypes of cancer cells [20]. A recent study has suggested miR-638 was involved in mediating DNA damage repair processes and might act as a sensitizer in cancer chemotherapy [21]. As a result, miR-638 may enhance the chemotherapeutic efficacy of certain drugs and help to improve the chance of recovery from malignant tumors. Interestingly, we have previously found that miR-638 may be involved in the regulation of glucose metabolism in colorectal cancer by targeting HIF-1α and PGK1. However, more research is warranted to for further investigation. Little is known of the effect of circulating exosomal miR-638 in colon carcinogenesis, cancer cells metabolism, and chemosensitivity analysis. Findings in the present study have provided strong evidence for the prognostic role of serous exosomal miR-638 in colon cancer progression. In the future, we will focus on investigating the potential roles of circulating exosomal miR-638 in colon cancer cells metabolism and their sensitivity to cancer chemotherapy drugs.

In summary, the present study firstly shows strong evidence that serous exosomal miR-638 can be a useful biomarker predicting prognosis of colon cancer. However, the underlying molecular mechanisms of circulating exosomal miR-638 in colon carcinogenesis and progression warrant to be elucidated in more future studies. We will be committed to the research of exosomal miR-638 as a promising molecular targeted drug for patients with colon cancer.

## MATERIALS AND METHODS

### Study design

This retrospective study was performed in colon cancer patients histologically diagnosed with the time of postoperative follow-up between 2008 and 2014 in our hospital. The mean follow-up period was 47 months (range 10-60). Outcomes of a total of 192 patients with colon cancer were identified and included into the present study. 39 healthy controls were randomly selected from individuals receiving health examination in our hospital. The study was approved by our hospital's ethical committee. Participants in this study had all signed the informed consent. The clinical and pathological data mainly included date of diagnosis, age, residential area, race, gender, tumor stages, tumor size, adjuvant chemotherapy, serum alkaline phosphatase, tumor differentiation status, and vascular infiltration. Patients were staged by the TNM classification of the International Union against Cancer. Peripheral blood samples were collected from each patient before surgery.

### Purification of exosomes from serum and extraction of total RNAs

Serous samples were separated from fresh peripheral blood by centrifugation at 1,200 *g* for 10 min at 4°C. The exosomes of plasma were purified by use of a total exosome isolation kit (Invitrogen, USA) based on the manufacturers’ protocol. Precipitations of exosomes were fully lysed by Trizol LS (Invitrogen Life Technologies, Paisley, UK) for subsequent RNA extraction. Total exosomal RNAs were extracted according to the protocol of miRNeasy mini kit (Qiagen, Netherlands). In brief, serum exosomes were diluted with 1 ml of Lysis Reagent. Subsequent extraction was carried out based on the manufacturer's protocol. The quality of total RNAs was assayed by an Agilent 2100 Bioanalyzer (Agilent Technologies, USA). Total RNAs were stored at −80°C for quantitative real-time PCR detection.

### Quantitative real-time PCR for serous exosomal miR-638

The expression of miR-638 in serum exosomes was detected using quantitative real-time PCR. Briefly, cDNA was synthesized from total RNAs using a PrimeScriptTM RT reagent Kit (Takara, Tianjin, China) according to the manufacturer's protocol. MicroRNA-16a was used as an internal control for real-time PCR analysis. Each sample was analyzed in duplicate. 2^−ΔΔCT^ was calculated to determine the relative expression of miR-638 in serum exosomes of patients with colon cancer.

### Outcome estimation

We investigated the association between levels of serum exosomal miR-638 and colon cancer outcome of overall survival (OS) and disease-free survival (DFS) by use of the Kaplan-Meier survival curve method. We carried out the follow-up every three months within the first three years and every six months after then.

### Statistical analysis

Data were shown as mean ± SEM. In this study, we used independent-samples T test or one-way ANOVA for statistical analysis. The association between levels of serum exosomal miR-638 and colon cancer outcome of OS and DFS was evaluated by use of Kaplan-Meier and log-rank tests. Hazard ratio (HR) with 95 % confidence interval (95 % CI) was used to determine the relative risk of mortality, which was calculated through the Cox regression analysis. Covariates used in the multivariate analysis mainly comprised of age, gender, tumor stage, tumor differentiation status, vascular filtration and levels of serum exosomal miR-638. Softwares of SPSS (version 16.0), Graphpad (version 5.0), and STATA (version 12.0) were used for statistical analysis.

## References

[1] Sideris M, Papagrigoriadis S (2014). Molecular biomarkers and classification models in the evaluation of the prognosis of colorectal cancer. Anticancer Res.

[2] Sameer AS, Nissar S (2016). Epigenetics in diagnosis of colorectal cancer. Mol Biol Res Commun.

[3] Tanaka T, Tanaka M, Ishigamori R (2010). Biomarkers for colorectal cancer. Int J Mol Sci.

[4] Cunningham D, Atkin W, Lenz HJ, Lynch HT, Minsky B, Nordlinger B, Starling N (2010). Colorectal cancer. Lancet.

[5] Audisio RA, Papamichael D (2012). Treatment of colorectal cancer in older patients. Nat Rev Gastroenterol Hepatol.

[6] Ogata-Kawata H, Izumiya M, Kurioka D, Honma Y, Yamada Y, Furuta K, Gunji T, Ohta H, Okamoto H, Sonoda H, Watanabe M, Nakagama H, Yokota J (2014). Circulating exosomal microRNAs as biomarkers of colon cancer. PLoS One.

[7] Hosseini M, Khatamianfar S, Hassanian SM, Nedaeinia R, Shafiee M, Maftouh M, Ghayour-Mobarhan M, Sales SS, Avan A (2017). Exosome-encapsulated microRNAs as potential circulating biomarkers in colon cancer. Curr Pharm Des.

[8] Mohammadi A, Mansoori B, Baradaran B (2016). The role of microRNAs in colorectal cancer. Biomed Pharmacother.

[9] Hausser J, Zavolan M (2014). Identification and consequences of miRNA-target interactions—beyond repression of gene expression. Nat Rev Genet.

[10] Melo SA, Sugimoto H, O'Connell JT, Kato N, Villanueva A, Vidal A, Qiu L, Vitkin E, Perelman LT, Melo CA, Lucci A, Ivan C, Calin GA, Kalluri R (2014). Cancer exosomes perform cell-independent microRNA biogenesis and promote tumorigenesis. Cancer Cell.

[11] Monzo M, Santasusagna S, Moreno I, Martinez F, Hernandez R, Munoz C, Castellano JJ, Moreno J, Navarro A (2017). Exosomal microRNAs isolated from plasma of mesenteric veins linked to liver metastases in resected patients with colon cancer. Oncotarget.

[12] Guo L, Guo N (2015). Exosomes: potent regulators of tumor malignancy and potential bio-tools in clinical application. Crit Rev Oncol Hematol.

[13] Milane L, Singh A, Mattheolabakis G, Suresh M, Amiji MM (2015). Exosome mediated communication within the tumor microenvironment. J Control Release.

[14] Anastasiadou E, Slack FJ (2014). Cancer. Malicious exosomes. Science.

[15] Soung YH, Nguyen T, Cao H, Lee J, Chung J (2016). Emerging roles of exosomes in cancer invasion and metastasis. BMB Rep.

[16] Cheng J, Chen Y, Zhao P, Liu X, Dong J, Li J, Huang C, Wu R, Lv Y (2016). Downregulation of miRNA-638 promotes angiogenesis and growth of hepatocellular carcinoma by targeting VEGF. Oncotarget.

[17] Zhang J, Bian Z, Zhou J, Song M, Liu Z, Feng Y, Zhe L, Zhang B, Yin Y, Huang Z (2015). MicroRNA-638 inhibits cell proliferation by targeting phospholipase D1 in human gastric carcinoma. Protein Cell.

[18] Zhang J, Fei B, Wang Q, Song M, Yin Y, Zhang B, Ni S, Guo W, Bian Z, Quan C, Liu Z, Wang Y, Yu J (2014). MicroRNA-638 inhibits cell proliferation, invasion and regulates cell cycle by targeting tetraspanin 1 in human colorectal carcinoma. Oncotarget.

[19] Lin Y, Zeng Y, Zhang F, Xue L, Huang Z, Li W, Guo M (2013). Characterization of microRNA expression profiles and the discovery of novel microRNAs involved in cancer during human embryonic development. PLoS One.

[20] Ren Y, Chen Y, Liang X, Lu Y, Pan W, Yang M (2017). MiRNA-638 promotes autophagy and malignant phenotypes of cancer cells via directly suppressing DACT3. Cancer Lett.

[21] He M, Lin Y, Tang Y, Liu Y, Zhou W, Li C, Sun G, Guo M (2016). miR-638 suppresses DNA damage repair by targeting SMC1A expression in terminally differentiated cells. Aging (Albany NY).

